# Effect of label elements in bottled water: Impact on consumer preferences, purchase intentions and health perception through affective sensory tests

**DOI:** 10.1016/j.heliyon.2024.e35106

**Published:** 2024-07-26

**Authors:** Reynaldo J. Silva-Paz, Tito A. Prada-Linarez, Thalia A. Rivera-Ashqui, Carmen R. Apaza-Humerez, Amparo Eccoña-Sota, Hernán E. Verde-Lujan

**Affiliations:** a Escuela Profesional de Ingeniería en Industrias Alimentarias, Departamento de Ingeniería, Universidad Nacional de Barranca, Av. Toribio de Luzuriaga N° 376 Mz J- Urb. La Florida, Barranca, Lima, Peru; b Escuela Profesional de Ingeniería de Industrias Alimentarias, Facultad de Ingeniería y Arquitectura, Universidad Peruana Unión, Carretera Central Km 19.5 Ñaña, Chosica, Lima, Peru

**Keywords:** Bottled water, Label, Eye tracker, Purchase intention, Preference

## Abstract

Bottled water has become a popular beverage choice worldwide, with consumers increasingly seeking healthier options. However, label elements can significantly influence consumer perception and purchasing decisions. The research aimed to assess how label elements affect the liking, purchase intention, preference and concept of healthy bottled water. Two stages involved 180 and 100 participants aged between 18 and 40, provided sociodemographic information. The first stage used a hedonic scale and ranking test to perception of nine labels with different elements. The second stage selected a consensus label from prior tests. Four labels were designed, differing in brand color and nutritional information placement. In this last stage, the acceptability, preference ranking and concept of healthy were re-evaluated and eye tracking via the Pupil Lab program. Findings showed varied responses in acceptability and purchase intention among consumers. However, significant differences were observed in preferences and healthiness perceptions based on label characteristics. The label with the highest preference and perceived healthiness featured a sky-blue design with nutritional information on the right side. Combining sensory testing and eye tracking offers valuable insights for designing labels that positively impact consumer perception. The results provide important implications for bottled water manufacturers and marketers in developing effective labeling strategies to meet consumer preferences and promote healthier choices.

## Highlights

• In the first stage, differences were identified between the design, information and presentation characteristics.
• In the second stage, it was found that the sky blue label with nutritional information on the right had greater preference and perception of healthiness.
• The most notable elements were the wave figure and the product brand.

## Graphical abstract

**Figure:**
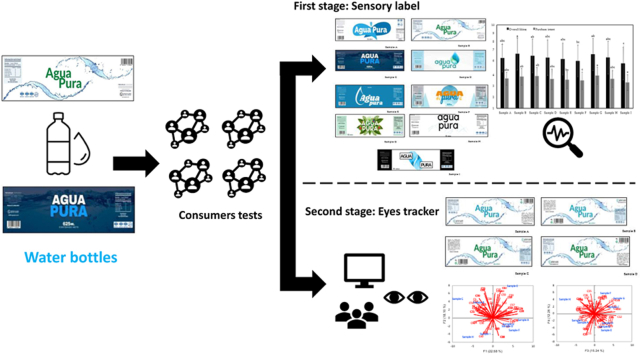

## 1 Introduction

Currently, consumers are increasingly oriented towards the search for products that meet high standards of safety, quality, and, that adaptability to their health needs and preferences. However, widespread concern arises due to the scarcity of details on labels regarding the ingredients used, nutritional composition, certifications, and other relevant characteristics. This lack of information limits consumers' ability to adequately evaluate the quality and suitability of the products they choose. In this sense, the information provided on the label plays a crucial role, as it has a significant influence on the purchasing decision-making process [1,2]. A product label plays a fundamental role in transmitting relevant and meaningful information to consumers [3]. It is particularly crucial in the case of the sale of water for human consumption, where the elements present on the label have a significant impact on the quality intention and general perception of consumers. Through the label, essential data is provided about the composition of the water, its origin, the treatment and purification processes, as well as the presence of possible additives or contaminants. These elements exert a direct influence on the purchasing decisions of consumers, who seek to ensure that the water they are purchasing is safe, of quality and suitable for their well-being and health [4,5].

Water consumption is essential to maintain optimal health and proper functioning of the body. Water plays a fundamental role in a wide range of vital physiological functions, including the regulation of body temperature, cellular hydration, the elimination of toxins and the transport of nutrients [5,6]. In addition, it is essential for the correct functioning of the systems and organs of the human body, such as the cardiovascular, digestive and renal systems. In the context of the sale of water for human consumption, various products are offered, ranging from bottles of bottled water to public filtered water dispensers. In many countries, including Peru, rigorous regulations and standards are established to guarantee the quality and safety of water supplied to the public. These standards include extensive quality testing, rigorous purification processes, and labeling requirements that provide detailed information to consumers about the origin and composition of the water they are purchasing [[7], [8], [9]].

Through labeling, the Food Industry seeks to promote purchases, stimulate brand loyalty, and provide consumers with information about the health and safety aspects of their products, partly also due to policy requirements [10]. The label of a product plays a fundamental role in conveying relevant and meaningful information to consumers [3]. For example, consumers perceive locally sourced food products as of better quality, safer, more environmentally friendly, and superior to those from elsewhere; furthermore, they feel it is their duty to support local or national production [11]. Consumer preference for labels stems from an interest in healthiness, origin, ingredients, among other factors; however, they do not read most elements of the labels, thus, their impact on consumer choices could be relatively small [10]. Labeling with graphic warnings, for example, can help consumers understand nutritional quality [12]. Despite the importance of label elements in influencing consumer perception and purchase intention, there is a lack of research specifically focused on bottled water. Most studies have examined the impact of labels on other food products, such as packaged goods or fresh produce, but have not delved deeply into the unique characteristics and consumer preferences related to bottled water. This gap in the literature limits the ability of manufacturers and marketers to develop effective labeling strategies tailored to the bottled water market.

Purchase intention is a central concept in the study of consumer behavior, which refers to a person's willingness or willingness to purchase a product or service. This decision-making process is influenced by a wide range of factors, including individual needs, product characteristics, previous purchasing experiences, third-party recommendations, and value perception [13]. That is, the purchase intention precedes the actual purchasing action, varying in intensity and duration depending on the situation and the product under consideration [14,15]. The study of purchase intention is of great relevance for both companies and market researchers. Understanding the motivations and factors that influence product procurement, allowing companies to design more effective marketing strategies adapted to the needs of consumers. In addition, analysis of purchase intention provides valuable information about consumer preferences and helps identify market opportunities. On the other hand, researchers use product acquisition data as an indicator to predict actual purchasing behavior and evaluate the effectiveness of different variables or stimuli in the consumer's decision-making process [16].

The use of the eye tracker in the analysis of visual elements offers a precise and objective measurement of consumers' eye movements. This valuable tool allows for the reliable and detailed identification of the areas that capture the most visual attention, the duration of fixation on each area, and the sequence in which they are explored. These data are essential to understand which visual elements, such as logos, certification seals or colors, attract consumers' attention and have an impact on their purchase intention, perception of quality and assessment of the health of the product [17,18]. In contrast to traditional methods such as questionnaires or interviews, which rely on consumers' memory and subjective perception, the eye tracker provides objective and detailed measurements of visual attention. Thanks to this tool, precise information is obtained about the direction of gaze and the visual elements that generate a greater ocular response [19]. This gives marketers the ability to precisely identify and adjust the visual aspects that influence purchase intent, thereby improving ad effectiveness and optimizing the consumer experience. By using the eye tracker, a deeper understanding of how consumers visually interact with the elements of the label is achieved, allowing informed and strategic decisions to be made to positively impact purchasing decision making and the perception of quality and health of the product. product [[20], [21], [22]]. This study provides a significant contribution to the field of consumer behavior and food product marketing. By focusing on the detailed analysis of how label elements influence purchase intention, quality perception, and the notion of a product being healthy. The findings of this study can assist companies in better understanding consumer preferences and perceptions. This, in turn, can enable them to optimize their labels and marketing messages to increase sales and brand loyalty. The general objective of this research work is to determine how the label elements influence the purchase intention, acceptability, preference and healthy concept of bottled water. In the first stage, (i) the perception of nine labels was analyzed based on acceptability, purchase intention and healthy concept of bottled water, to select the label to be used in the next stage. The second stage (ii) consisted of evaluating the time and number of fixations of the label elements using a four-label eye tracker, varying the color and position. Additionally, know the acceptability, purchase intention and healthy concept to select the most appropriate label for bottled water.

## 2 Materials and methods

### 2.1 Participants

We worked with 100 and 80 participants in stages 1 and 2, respectively. The consumers were university students and teachers from the Faculty of Engineering and Architecture of the Universidad Peruana Unión, aged between 18 and 40 years. Each of them participated after giving their informed consent, having the freedom to withdraw from the test at any time they considered appropriate. Participants reported consuming bottled water regularly. In addition, they indicated that they did not have audiovisual or health problems. The trials were carried out between February–March 2023.

### 2.2 Stimulus

**Fig. 1:**
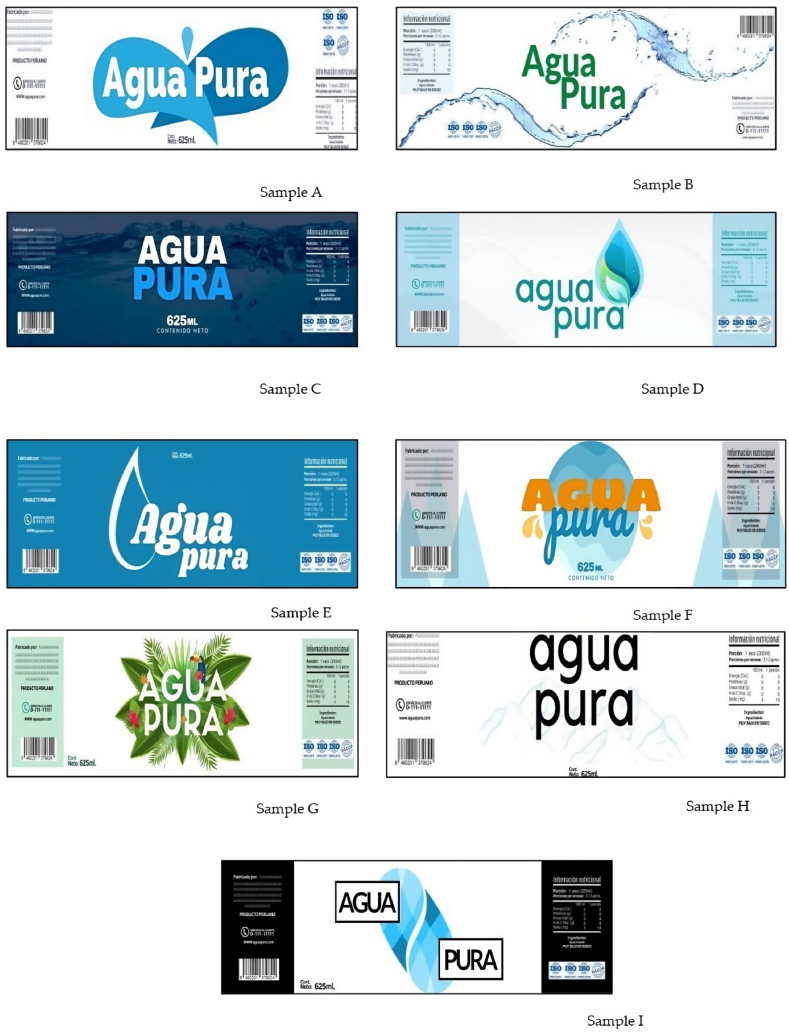
Water labels used in the first evaluation stage.

Nine images of water packets were used, each on an A4 printed sheet. These visual stimuli were created using Autodesk 3ds Max Design 2011, and presented two variables in packaging design: color and format (Fig. 1). The colors chosen were a gray scale, cold colors and warm colors. The formats that will be used are water labels with different elements: sensory message, color and size. The images did not contain references to actual trademarks or brand names, as respondents might perceive certain product attributes of a trademark. The fictitious brand “Pure Water” (“Agua Pura”, Spanish language) was used in all images. This brand was chosen as its simplicity meant it would not interfere with the study and the designs were based on national bottled water labels.

### 2.3 Eye trackers

The equipment used was the Eye Tracker Pupil Lab 3.60, it had a desktop computer and an 18.5” LED monitor, with a screen resolution of 1366-768 pixels. All of these devices were connected to each other. The Eye Tracker had a sampling rate of 60 Hz and a precision level of 0.5°. Additionally, Pupil Lab software was used to present the stimuli, calibrate the eye tracker, record data related to participants' eye movements and fixation behavior, and form statistical data. The participants were placed in an ergonomic chair at a distance of 30 cm from the monitor, the environment was conditioned to avoid strange noises.

### 2.4 Experimental methodology

Fig. 1 shows the different images of the water labels that will be presented to the participants and a questionnaire to evaluate them. To do this, they spent an average time of approximately 30 min completing the survey, during which the researchers were present to answer any questions or queries. Once the questionnaires were completed, participants received a package of cookies to thank them for helping in the study.

The survey in the first and second stages evaluated a total of five items: (i) Sociodemographic data, (ii) acceptability, (iii) purchase intention, (iv) preference and (v) healthy concept. The sociodemographic data comprised a series of questions to determine the profile of the respondent (age, sex and educational level). In the first stage, they were asked to record their sociodemographic data, then respondents were asked to rate the acceptability with a 9-point hedonic scale (1 = I dislike it very much and 9 = I like it very much), in terms of purchase intention. We worked with a 5-point scale (1 = I would not buy it and 5 = Yes, I would buy it), in the preference and healthy concept a ranking scale was used, where the samples were placed from lowest to highest preference or healthy, respectively. They had the option to leave questions blank if they could not answer. The evaluation slip explained that the different packages contained the same amount of water and would have the same price (although the price was not specified). To understand the perception of these labels and the elements that attract consumers, the experiments were carried out in a quiet environment. In the second stage, the label that jointly presented greater acceptability, purchase intention, preference and healthy concept was selected. From this label, four labels were designed that differed in the color of the brand and location of the nutritional information. Labels were presented in a monadic form to evaluate the acceptability and purchase intention of each of them. Then they were presented with the four labels together so that they could perform a preference ranking test based on what they considered healthier (from lowest to highest). These tests were carried out using an evaluation sheet and the use of eye trackers.

### 2.5 Statistical analysis

Demographic data were analyzed in terms of selection frequency. A repeated measure analysis of variance (ANOVA) was performed on the data to verify the effect of labels with respect to acceptability and purchase intention. Significant differences were calculated using the Tukey test. The differences were considered significant when p < 0.05 [23]. On the other hand, the Friedman test was applied for the preference ranking and healthy concept test. If differences were found, the corrected Bonferroni test was used [24]. Additionally, the data were subjected to principal component analysis (PCA) to explore underlying patterns and relationships between variables. PCA was conducted to reduce the dimensionality of the data set and identify key components of acceptability and purchase intention that contribute to consumer behavior and perception [25]. The following areas of interest (AOI) were defined on the labels: brand size, manufacturer, best before date, net content, nutritional information, recommendation and health claim. For each AOI, measurements were calculated using the eye-tracking software: number and time of consumers' fixations where they fixate their gaze on the AOI (minimum fixation time was 100 ms). All data analyses were performed using XLSTAT 2023 software [26].

## 3 Results

### 3.1 Sociodemographic data of the participants in stage I and II

Table 1 presents the sociodemographic data of the participants. In the first stage there were 100 consumers and in the second stage there were 80 participants. In both cases, a balance (50 %) was observed between male and female participants, although in the second stage the participation of women was slightly higher (>60 %). Regarding the age of the participants, more than 80 % of the participants are between 18 and 30 years old. Consumers were mostly from the coastal region, followed by the mountains and to a lesser extent the jungle. The socioeconomic level of the participants was normally between the medium level (>40 %) and low (>20 %). These data reflect the current situation of the country, where the majority of the population belongs to these socioeconomic levels.

**Table 1:** Sociodemographic data report for stage 1 and 2.

Data	Stage 1		Stage 2	
	n	%	n	%
Gender				
Female	51	51.00	50	62.50
Male	49	49.00	30	37.50
Age				
18–30	83	83.00	79	98.75
31–40	11	11.00	1	1.25
>41	6	6.00	0	0.00
Place of origin				
Coast	56	56.00	54	67.50
Mountain range	20	20.00	18	22.50
Jungle	24	24.00	8	10.00
Socioeconomic level				
Low	28	28.00	22	27.50
Half	46	46.00	44	55.00
High	16	16.00	12	15.00

#### 3.1.1 Stage 1

##### 3.1.1.1 Sensory perception of the different labels

**Fig. 2:**
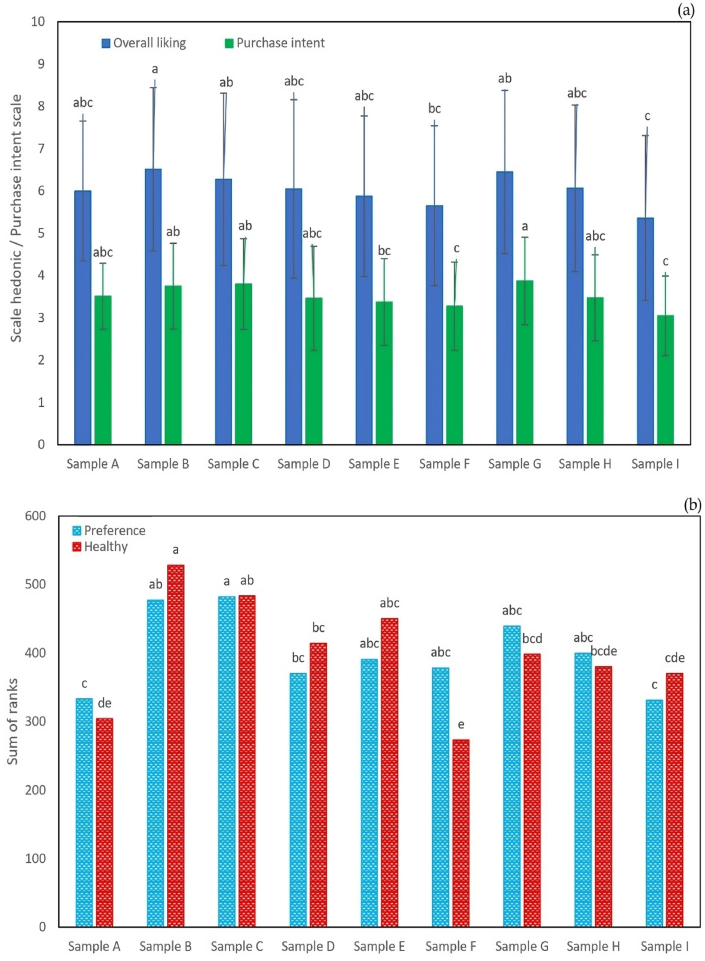
Chart of acceptability and purchase intention (a) and preference and health concept of ranking test (b).

The results of the first stage are seen in Fig. 2(a) and (b). Participants were presented with nine labels to evaluate their acceptability and purchase intention.

Regarding acceptability, all samples showed values between 6 and 9 ("I like it" to "I like it very much"), except samples I, which indicated that "I neither like it nor dislike it", that is, a neutral perception, this behavior was similar for sample F and H (p > 0.05) which did not present significant differences with sample I. Purchase intention, all labels were rated as “I would probably buy it”, however, significant differences were found (p < 0.05) between labels. Samples F, H and I obtained the lowest scores, but similar to each other. Finally, the sorting test was carried out by preference and by healthy label concept. Based on preference, consumers indicated sample B, although statistically it is similar to C, E, F, G and H. The lowest preference was obtained by sample A and I. Regarding health, a behavior was observed similar. Sample B showed a higher score, although it is not significantly different from C and E, the samples perceived as less healthy were A, F, H and I.

Table 2 presents the results of the Principal Component Analysis (PCA) conducted on the samples, with consumer acceptability and purchase intention scores represented across four principal components (F1, F2, F3, F4). The position of each sample in the sensory map of the PCA space is determined by its scores on these components [27]. Higher scores indicate a greater association with that component, and vice versa [25]. Overall, samples A, E, and F show positive associations with various components for both acceptability and purchase intention, while samples B, C, H, and I exhibit a variety of positive and negative associations with the components, either in acceptability or purchase intention. Sample C demonstrates a positive association with F2 and F3 in terms of acceptability, suggesting that evaluators tend to have a favorable opinion of this sample in relation to these dimensions. However, in terms of purchase intention, sample C has a positive association with F2 and a negative association with F3, indicating a greater willingness to purchase in relation to F2 but a lesser willingness in relation to F3. Samples D, G, and H have mixed associations in terms of liking and purchase intention across different components.

**Table 2:** Position of sample in space sensory apply component principal analysis.

Sample	Liking			
	F1	F2	F3	F4
Sample A	5.85091655	−0.063176	−2.23194597	−2.08904426
Sample B	−5.36082589	−5.28606024	1.06721773	−3.32929446
Sample C	−7.78715439	4.04285382	3.2390052	1.13591932
Sample D	1.88520401	−1.70017445	1.54792329	−2.71268669
Sample E	3.19049221	7.24308561	1.62016928	−3.46406007
Sample F	3.64184496	−2.68144604	1.13822738	5.03899684
Sample G	2.13941937	−1.1593071	4.62056078	3.63744018
Sample H	−2.51037044	3.12096207	−7.07732816	3.33750525
Sample I	−1.04952639	−3.51673766	−3.92382954	−1.55477611
**Purchase intention**				
Sample A	11.5472162	10.862902	14.366509	5.25306179
Sample B	4.20384585	0.13488491	39.5437903	7.30450824
Sample C	0.09633194	60.9271643	1.16645567	15.482772
Sample D	5.27197532	2.79017347	1.37034127	0.04882893
Sample E	0.72614139	8.42103404	32.9683449	1.47422656
Sample F	32.9729942	2.30480376	2.70910707	2.58876856
Sample G	6.97327357	1.26582499	0.90369702	7.77369942
Sample H	37.497983	0.03114449	1.63988445	19.9583819
Sample I	0.71023851	13.2620681	5.33187022	40.1157527

**Fig. 3:**
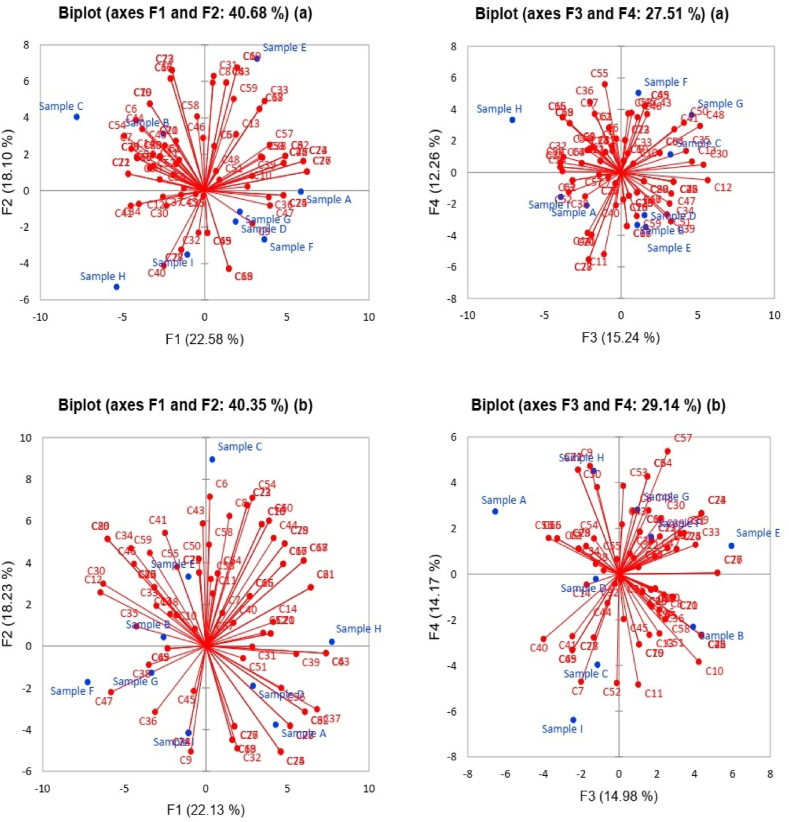
Graph of principal components analysis of consumers regarding acceptability (a) and purchase intention (b).

Fig. 3 shows consumer responses based on their acceptability (Fig. 3(a)) and purchase intention (Fig. 3(b)) through a principal component analysis. Graphs with three dimensions were made to explain 68.19 and 69.49 % of the total variability of the data for acceptability and purchase intention, respectively. Regarding acceptability, it is observed that consumers are very variable in the acceptability of the samples, there is no homogeneity in their response. A segmentation of four groups can be observed, the first group made up of samples B and C, the second group shows E, the third group samples H and I, and the fourth group samples A, D, G and F, in the first two dimensions. However, when describing the first and third dimensions, there is a similar behavior, although the location of the samples varies. Regarding purchase intention, in the first two dimensions the formation of six groups is presented, first group sample C, second group sample H, third group sample I, fourth group samples D and A, fifth group samples G and F, and sixth group samples B and E. The first and third dimensions show the same results varying the location of the samples. Based on the results of the first stage, label B is selected, as it presents slightly higher values in acceptability, purchase intention, ranking by preference and healthy product concept.

#### 3.1.2 Stage 2

##### 3.1.2.1 Acceptability and purchase intention of labels

**Fig. 4:**
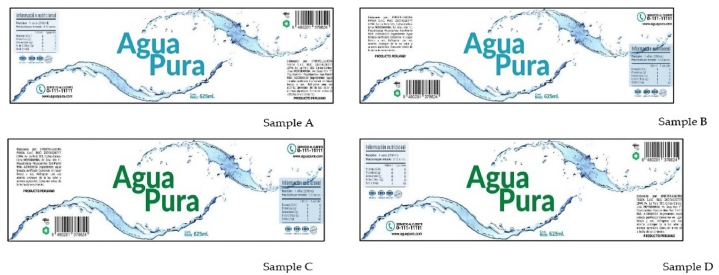
Label models used for the second stage.

**Fig. 5:**
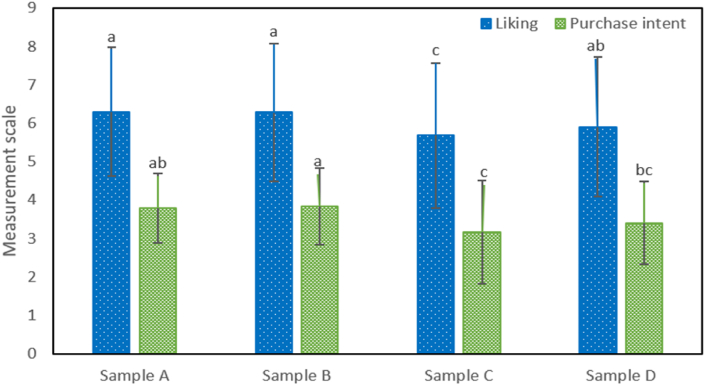
Acceptability and purchase intention of the different labels.

Fig. 4 presents the labels with the different elements used in the second session. In addition, the color of the brand and location of the nutritional information were varied. Fig. 5 shows the results of acceptability and purchase intention, which show significant differences (p < 0.05) in the water labels. Regarding acceptability, sample A, B and D did not present significant differences, being similar between them, although they are all different from sample C, which had a lower rating. A similar behavior was observed in purchase intention. Where samples A and B had greater purchase intention. Through the analysis of variance, it was found that acceptability has a significant influence by color (p = 0.032), however, the position of the information (p = 0.528) and the color-position of information interaction (p = 0.665) do not influence the purchase intention. , a similar behavior was evident, the color was statistically significant (p = 0.047) although not in the position of the information (p = 0.669) and the color-position of information interaction (0.454).

##### 3.1.2.2 Ranking test by consumer preference and healthy label

Table 3 presents the results of the ranking test by preference and healthy label, an interesting effect is observed, where although the number and total time of fixations of the labels with respect to the healthy label did not present significant differences Through eye-tracking, the sorting test showed significant differences where samples A and B are considered healthier than the rest of the samples, that is, the color of the label influences this decision. Regarding preference, the labels showed significant differences (p < 0.05), label B was selected as the one with the highest preference compared to the rest of the samples, despite the fact that the average fixation duration time did not change significantly.

**Table 3:** Results of the preference and health ranking test on labels.

Preference			Healthy	
Sample	Sum of ranks	Mean of ranks	Sum of ranks	Mean of ranks
Sample A	10675	152.5 ^b^	10877	155.386 ^bc^
Sample B	13265	189.5^c^	11745	167.785^c^
Sample C	7035	100.5^a^	7451	106.442^a^
Sample D	8365	119.5^a^	9267	132.384 ^b^

##### 3.1.2.3 Evaluation of times and numbers of fixations of label applying liking, healthy and preference ranking test using eye trackers

The influence of the label design (Fig. 4) on the number and duration of fixations can be seen in Table 4. Consumers made fewer fixations during a shorter period of time when evaluating each of the label's B and D individually, these samples did not present significant differences between them. Label C showed higher values in the number of fixations, although the fixation time was similar to samples A and B. Consumers made an average of 1914.24 ms of fixations to evaluate the labels. On the other hand, when evaluating the perceived elements of the products, consumers made an average number of fixations of 11.47. As shown, the differences between the label elements did not present significant differences for both the number and fixation time (p > 0.05) in the case of general information, barcode, customer service logo, net content logo and the assigned random code. However, significant differences (p < 0.05) were evident between the wave figure and the label mark in both response variables.

**Table 4:** Fixation time and numbers based on eye-tracking measurements applied to the liking, preference ranking test and healthy label calculated on the defined areas of interest.

Parameters	Liking		Healthy		Preference	
	n. fix FRONT	t (ms) FRONT	n. fix FRONT	t (ms) FRONT	n. fix FRONT	t (ms) FRONT
**Sample**						
Sample A	12.109 ^ab^	2125.094^a^	3.402^a^	536.576^a^	3.474^a^	1609.399^a^
Sample B	10.230 ^b^	1761.503 ^ab^	3.556^a^	542.136^a^	1.993 ^ab^	1058.320^a^
Sample C	13.546^a^	2149.855^a^	2.735^a^	423.192^a^	1.035 ^b^	925.922^a^
Sample D	10.032 ^b^	1626.518 ^b^	4.097^a^	638.156^a^	0.431 ^b^	387.224^a^
**Factor (p-value)**						
Color	0.038	0.026	0.537	0.574	0.041	0.046
Position	0.575	0.981	0.581	0.668	0.247	0.262
Color x Position	0.226	0.137	0.664	0.600	0.662	0.665
**Areas of interest (AOI)**						
Wave figure	68.661^a^	11676.149^a^	21.392^a^	3321.365^a^	10.658^a^	4484.101^a^
Brand	18.729 ^b^	2982.136 ^b^	2.818 ^b^	437.148 ^b^	1.626 ^b^	2371.665 ^ab^
General information	1.843^c^	278.109^c^	1.983 ^b^	304.480 ^b^	1.083 ^b^	192.033 ^b^
Barcode	1.075^c^	159.460^c^	0.649 ^b^	101.165 ^b^	0.361 ^b^	76.632 ^b^
Customer Support	0.814^c^	119.211^c^	0.467 ^b^	77.357 ^b^	0.293 ^b^	42.853 ^b^
Logo	0.300^c^	46.481^c^	0.155 ^b^	22.156 ^b^	0.090 ^b^	14.010 ^b^
Net content	0.236^c^	37.785^c^	0.077 ^b^	11.494 ^b^	0.040 ^b^	6.517 ^b^

Regarding the number and time of fixation, as shown in Table 4, the number and time of fixations differed significantly depending on the question asked. When asked about the idea of how healthy a label is, there were no significant differences (p > 0.05) in the time and number of fixations. When asking which of the samples you prefer, it was observed that the fixation time did not present significant differences between the labels, although the number of fixations in sample A and C presented higher values than samples B and D, although sample B was statistically similar. Color significantly influences (p > 0.05) the acceptability and preferences for both the number and fixation time, the position and the color-position of information interaction did not influence the response variables studied. Regarding the area of interest considered, a similar behavior was observed based on the preference and idea of healthy questions. In all cases, the wave figure was significantly different from the rest of the elements, both for the time and number of fixations, and for brand in the health perception task, then for the rest of the areas of interest (Fig. 4).

Evaluation type (question) and the interaction between area of interest and evaluation type also significantly affected the average number of fixations, with people looking longer when asked about the healthiest label (Table 2). According to Jacob and Karn [28], fixation count or number of fixations is related to information processing and the importance of information to consumers.

## 4 Discussion

### 4.1 Stage 1

#### 4.1.1 Sensory perception of the different labels

Consumers often have certain preferences regarding the information they want to find on bottled water labels. Some elements that are usually of interest are the origin of the bottled water and its purity (if the water comes from a natural source or spring, it has been subjected to filtration or purification processes). In addition to nutritional information, although water itself does not contain calories or nutrients, consumers may value the inclusion of additional nutritional information on the label, especially if the water is fortified with minerals or other additives [29]. Consumers often look for information about container size, whether in volume or number of bottles, to make purchasing decisions based on their needs and preferences. Also, they look for additional characteristics in bottled water, such as alkaline water, with electrolytes, with vitamins or with natural flavors [30]. On the other hand, in recent years, consumer concern about the environmental impact of water containers has increased. Therefore, many consumers appreciate that the label includes information about sustainability, such as whether the packaging is recyclable, made from renewable materials, or whether the company has environmental responsibility policies [[31], [32], [33]]. Consumer associations regarding acceptability and purchase intention can be attributed to various factors, including intrinsic product characteristics, individual consumer perceptions [24], as well as external influences such as advertising and branding [1].

Regarding acceptability, it is observed that consumers are very variable in the acceptability of the samples, there is no homogeneity in their response. The first and third dimensions show the same results varying the location of the samples. This is attributed to the fact that a group of participants generates a first classification of the samples and in another dimension these classifications vary due to their preferences, due to a series of factors that influence their purchase intentions and acceptability. Consumers have different needs, wants and preferences. Each person has unique circumstances, personal values and priorities that influence their purchasing decisions. What may be important to one consumer may not be important to another. On the other hand, previous experiences with similar products can influence your responses and preferences. Past interactions with certain brands or products can create a positive or negative predisposition toward them. In addition, demographic factors such as age, gender, educational level, and cultural background may also influence responses and preferences. Different generations may have different approaches and values in relation to sustainability, health or convenience. Likewise, exposure to various social and cultural influences that can affect your responses and preferences. This includes the influence of family, friends, reference groups, advertising, current trends and social norms [1,[34], [35], [36], [37]].

### 4.2 Stage 2

#### 4.2.1 Acceptability and purchase intention of labels

Various research confirms that the label is a key factor that consumers rely on when evaluating and purchasing foods and beverages [[38], [39], [40]]. Zafar et al. [41] found that label information and its format influence consumers' attitudes and purchase intention. Our findings demonstrated that acceptability and purchase intention were influenced by the color and location of the information. Consumers accepted a light blue label with information located on the right, agreeing with Banović, Fontes, Barreira and Grunert [42] indicated that consumers evaluate novel products with greater emphasis through their extrinsic characteristics than through intrinsic sensory attributes. On the other hand, in a study of fermented drinks, the use of labels influenced the sensory perception of these drinks, because the opinion of consumers was heterogeneous (different sociodemographic and cultural characteristics, personal concepts and/or psychological aspects). and different expectations in relation to a product) [43].

#### 4.2.2 Ranking test by consumer preference and healthy label

It is important to know the preference of the product based on its label and to identify the healthy label by consumers, despite the fact that consumers indicate taste as their main interest when choosing food products. The label of healthy foods mainly emphasizes health attributes (nutritional information, list of ingredients, sensory messages such as low-calorie content, reductions in fat or sugar) rather than taste [44]. In the study, label B (light blue color and nutritional information on the right) was selected as the most preferred and healthy product, managing to relate a product focused on healthy and sensory choices, compared to the rest of the samples. According to Radach et al. [45] visual processing of print advertisements is characterized by a rapid phase of global processing, followed by a detailed scan of selected areas containing relevant information. According to the results of the present study, consumer visual processing of labels was quite similar, but focused primarily on selected pieces of information that are considered relevant. Most consumers searched for specific information they extracted through viewing.

#### 4.2.3 Evaluation of times and numbers of fixations of label applying liking, healthy and preference ranking test using eye trackers

By investigating which label elements attract your attention we can improve food development and innovation. The duration and number of fixations are measuring that eye tracking allows to study cognition and attention [28,46]. These measures reflect how visual information is processed [47]. Longer fixation duration is related to more detailed cognitive processing and careful analysis of information [48], and suggests complexity, interest, or engagement [49]. Regarding the number of fixations, it was significant for acceptability and preference, although for fixation time it was significant only for acceptability. Participants are more likely to observe different labels with greater variation when asked about acceptability and preference. During the evaluation of the description of the healthy product, no significant differences were found, because the colors used do not produce novel or familiar concepts. This is consistent with the findings of Garber et al. [50] who indicate that the color green is related to healthy/organic/ecological products, while the color black is related to luxury/premium products. Several studies have analyzed how food labels influence consumers' purchasing decisions. It was observed that consumers pay attention to the brand, nutrition, ingredients and image on labels to evaluate health perception and willingness to purchase. The density of information on labels can affect consumer attention, highlighting the importance of graphics that convey health information. Furthermore, they have shown that attributes such as product name and brand are crucial in the visual processing of labels, which can guide manufacturers in presenting information relevant to market success, especially in impulse purchases [[51], [52], [53], [54]].

## 5 Conclusions

This study has demonstrated that the use of labels on bottled water has a significant impact on consumer acceptability and purchase intention. Sensory tests and eye tracking revealed significant differences in consumer preferences and visual attention towards the labels, highlighting elements such as the wave pattern, brand logo, and nutritional information as most relevant. However, there was observed lack of uniformity in consumer responses, suggesting that preferences may vary depending on specific label characteristics. Eye tracking found significant differences in acceptability and preferences, although it did not reveal significant differences in the concept of health. These sensory tests and eye tracking provided valuable information on label elements that attract more attention. Despite its contributions, this study has some limitations, such as the lack of consideration of external factors such as cultural influence, sensory message on the label in the product. Nevertheless, these findings offer companies the opportunity to design more effective, informative, and attractive labels for their products, which could enhance the consumer experience, positively influence purchase intention, and strengthen brand loyalty.

## Limitation and implication

Our study has limitations in not fully considering external factors like cultural influences and sensory messaging on product labels, which could affect consumer perceptions. Sample size and demographics may influence generalizability. The implications suggest that companies can enhance consumer experience by strategically designing labels incorporating visually appealing and informative elements in shaping consumer perceptions and preferences in a competitive marketplace.

## Funding

The Universidad Nacional de Barranca provided funding for both the research and the Article Processing Charges (APC).

## Data availability statement

The data included in article and supplementary material in article.

## Ethics approval

The study was conducted in accordance with the ethical principles outlined in the Declaration of Helsinki. The research protocol was reviewed and approved by the Ethics Review Committee of the Faculty of Engineering and Architecture of the University Peruvian Union (0003-2023-CE-FIA). All subjects involved in the study provided informed verbal consent.

## CRediT authorship contribution statement

**Reynaldo J. Silva-Paz:** Writing – original draft, Methodology, Investigation, Formal analysis, Conceptualization. **Tito A. Prada-Linarez:** Resources, Methodology, Data curation, Conceptualization. **Thalia A. Rivera-Ashqui:** Visualization, Software, Resources, Methodology, Investigation, Data curation. **Carmen R. Apaza-Humerez:** Project administration, Methodology, Investigation, Funding acquisition. **Amparo Eccoña-Sota:** Writing – original draft, Software, Resources, Project administration, Investigation, Data curation. **Hernán E. Verde-Lujan:** Writing – review & editing, Validation, Supervision, Resources, Project administration, Funding acquisition.

## Declaration of competing interest

The authors declare that they have no known competing financial interests or personal relationships that could have appeared to influence the work reported in this paper.

## References

[1] Solomon M.R., Bamossy G., Askegaard S., Hogg M.K. (2016).

[2] Radner R., Smelser Neil J., Baltes Paul B. (2001). Bounded and Costly Rationality in International Encyclopedia of the Social & Behavioral Sciences.

[3] Bandara B.E.S., De Silva D.A.M., Maduwanthi B.C.H., Warunasinghe W.A.A.I. (2016). Impact of food labeling information on consumer purchasing decision: with special reference to faculty of Agricultural Sciences. Procedia Food Science.

[4] Martínez-de-Alegría I., Río R.M., Zarrabeitia E., Álvarez I. (2021). Heating demand as an energy performance indicator: a case study of buildings built under the passive house standard in Spain. Energy Pol..

[5] Villanueva C.M., Garfí M., Milà C., Olmos S., Ferrer I., Tonne C. (2021). Health and environmental impacts of drinking water choices in Barcelona, Spain: a modelling study. Sci. Total Environ..

[6] Schroeder E.D., Meyers Robert A. (2003). Encyclopedia of Physical Science and Technology.

[7] Mendez-Ruiz J.I., Barcia-Carreño M.B., Mejía-Bustamante L.J., Cornejo-Pozo Á.K., Salas-Vázquez C.A., Valverde-Armas P.E. (2023). Assessment of the performance of a water treatment plant in Ecuador: hydraulic resizing of the treatment units. Sustainability.

[8] Vázquez-García V. (2021). Gender, environmental disasters, and bottled water consumption: the case of the sonora river. Región Soc..

[9] Ferro P., Farfan-Solis R., Blanco-Shocosh D., Ferró-Gonzáles A.L., Ferro-Gonzales P.F. (2023). Determination of inorganic chemical parameters in drinking water in districts of the province of Puno in the region Puno-Peru. Heliyon.

[10] Meijer G.W., Detzel P., Grunert K.G., Robert M.C., Stancu V. (2021). Towards effective labelling of foods. An international perspective on safety and nutrition. Trends Food Sci. Technol..

[11] Thøgersen J. (2023). How does origin labelling on food packaging influence consumer product evaluation and choices? A systematic literature reviews. Food Pol..

[12] Pettigrew S., Jongenelis M., Maganja D., Hercberg S., Julia C. (2024). The ability of nutrition warning labels to improve understanding and choice outcomes among consumers demonstrating preferences for unhealthy foods. J. Acad. Nutr. Diet..

[13] Colet R., Polío J. (2014).

[14] Pérez A., del Bosque I.R. (2015). How customers construct corporate social responsibility images: testing the moderating role of demographic characteristics. BRQ Business Research Quarterly.

[15] Berki-Kiss D., Menrad K. (2022). The role emotions play in consumer intentions to make pro-social purchases in Germany–An augmented theory of planned behavior model. Sustain. Prod. Consum..

[16] Rana J., Paul J. (2017). Consumer behavior and purchase intention for organic food: a review and research agenda. J. Retailing Consum. Serv..

[17] Wedel M., Pieters R. (2008). A review of eye-tracking research in marketing. Rev. Market. Res..

[18] Khamis M., Hoesl A., Klimczak A., Reiss M., Alt F., Bulling A. Eyescout (2017). Proceedings of the 30th Annual ACM Symposium on User Interface Software and Technology.

[19] Jansson-Boyd Cathrine V., Bright Peter, Jansson-Boyd Cathrine V., Bright Peter (2024). Visual Neuroscience in Consumer Neuroscience.

[20] Carter B.T., Luke S.G. (2020). Best practices in eye tracking research. Int. J. Psychophysiol..

[21] Holmqvist K., Nyström M., Mulvey F. (2012). Proceedings of the Symposium on Eye Tracking Research and Applications.

[22] Niehorster D.C., Cornelissen T.H., Holmqvist K., Hooge I.T., Hessels R.S. (2018). What to expect from your remote eye-tracker when participants are unrestrained. Behav. Res. Methods.

[23] Piqueras-Fiszman B., Spence C. (2012). The influence of the color of the cup on consumers' perception of a hot beverage. J. Sensory Stud..

[24] Stone H., Bleibaum R.N., Thomas H.A. (2020).

[25] Naes T., Brockhoff P.B., Tomic O. (2010).

[26] Addinsoft (2023). XLSTAT statistical and data analysis solution.

[27] Hair J.F., Black W.C., Babin B.J., Anderson R.E. (2019).

[28] Jacob R.J., Karn K.S. (2003).

[29] Areebah Sohail, Maheen Amjad, Haseeb Munir, Danish Ahmed Siddiqui (July 2, 2020).

[30] Tilahun M., Beshaw M. (2020). Customer's perception and preference towards packaged drinking water. Sci. World J..

[31] Minton E.A., Rose R.L. (1997). The effects of environmental concern on environmentally friendly consumer behavior: an exploratory study. J. Bus. Res..

[32] Wansink B., Chandon P. (2006). Can "low-fat" nutrition labels lead to obesity?. J. Market. Res..

[33] Dekhili S., Akli Achabou M. (2014). Eco-labelling brand strategy: independent certification versus self-declaration. Eur. Bus. Rev..

[34] Schiffman L.G., Kanuk L.L., Wisenblit J. (2010).

[35] Verhoef P.C., Lemon K.N., Parasuraman A., Roggeveen A., Tsiros M., Schlesinger L.A. (2009). Customer experience creation: determinants, dynamics and management strategies. J. Retailing.

[36] Folkes V., Matta S. (2004). The effect of package shape on consumers' judgments of product volume: attention as a mental contaminant. J. Consum. Res..

[37] Cialdini R.B. (2009).

[38] Favier M., Celhay F., Pantin-Sohier G. (2019). Is less more or a bore? Package design simplicity and brand perception: an application to Champagne. J. Retailing Consum. Serv..

[39] Laeng B., Suegami T., Aminihajibashi S. (2016). Wine labels: an eye-tracking and pupillometry study. Int. J. Wine Bus. Res..

[40] Jaud D.A., Melnyk V. (2020). The effect of text-only versus text-and-image wine labels on liking, taste and purchase intentions. The mediating role of affective fluency. J. Retailing Consum. Serv..

[41] Zafar M.Z., Shi X., Yang H., Abbas J., Chen J. (2022). The impact of interpretive packaged food labels on consumer purchase intention: the comparative analysis of efficacy and inefficiency of food labels. Int. J. Environ. Res. Publ. Health.

[42] Banović M., Fontes M.A., Barreira M.M., Grunert K.G. (2012). Impact of product familiarity on beef quality perception. Agribusiness.

[43] S C.S.E., Zuim L., de Paula M.C., Mota M.F., Lima Filho T., Della Lucia S.M. (2021). The influence of musical song and package labeling on the acceptance and purchase intention of craft and industrial beers: a case study. Food Qual. Prefer..

[44] Braley Turnwald B.P., Crum A.J. (2019). Smart food policy for healthy food labeling: leading with taste, not healthiness, to shift consumption and enjoyment of healthy foods. Prev. Med..

[45] Radach Ralph, Hyönä Jukka, Deubel H. (2003). The mind's eye: cognitive and applied aspects of eye. Movement Research.

[46] Lai M.-L., Tsai M.-J., Yang F.-Y., Hsu C.-Y., Liu T.-C., Lee S.-W.-Y., Lee M.-S., Chiou G.-L., Liang J.-C., Tsai C.-C. (2013). A review of using eye-tracking technology in exploring learning from 2000 to 2012. Educ. Res. Rev..

[47] Motoki K., Saito T., Onuma T. (2021). Eye-tracking research on sensory and consumer science: a review, pitfalls and future directions. Food Res. Int..

[48] Glockner Glöckner A., Herbold A.-K. (2011). An eye-tracking study on information processing in risky decisions: evidence for compensatory strategies based on automatic processes. J. Behav. Decis. Making.

[49] Wang Q., Yang S., Liu M., Cao Z., Ma Q. (2014). An eye-tracking study of website complexity from cognitive load perspective. Decis. Support Syst..

[50] Garber Garber L.L., Burke R., Jones J. (2000).

[51] Ares Ares G., Giménez A., Bruzonne F., Vidal L., Antúnez L., Maiche A. (2013). Consumer visual processing of food labels: results from an eye-tracking study. J. Sensory Stud..

[52] Oliveira D., Machín L., Deliza R., Rosenthal A., Walter E., Giménez A., Ares G. (2016). Consumers' attention to functional food labels: insights from eye-tracking and change detection in a case study with probiotic milk. LWT-Food Sci. Technol..

[53] Rebollar R., Lidon I., Martin J., Puebla M. (2015). The identification of viewing patterns of chocolate snack packages using eye-tracking techniques. Food Qual. Prefer..

[54] Varela P., Antúnez L., Silva Cadena R., Giménez A., Ares G. (2014). Attentional capture and importance of package attributes for consumers' perceived similarities and differences among products: a case study with breakfast cereal packages. Food Res. Int..

