# Supplementary material for: Effect of label elements in bottled water: Impact on consumer preferences, purchase intentions and health perception through affective sensory tests
Source: Heliyon. 2024 Jul 26;10(15):e35106. doi: 10.1016/j.heliyon.2024.e35106 (PMC11336444; doi:10.1016/j.heliyon.2024.e35106)
Supplement: Multimedia component 1 [file mmc1.pdf]

Label evaluation report – Stage 1

Introduction:

welcome to this sensory evaluation of bottled water, in order to continue you must give your informed consent, remember that you can withdraw at any time you consider appropriate, any questions can be made to the person responsible for the sensory analysis (Reynaldo Silva-Paz or Tito Prada-Linares), wish to continue by accepting informed consent:

Yes ☐ No ☐

Indications:

- You will receive 09 water labels.
- Please look at the labels carefully and answer the following questions

Sample N°: __________

On a scale of 1 to 9, how much do you like this bottled water product?

I dislike it a lot I like it a lot

☐ ☐ ☐ ☐ ☐ ☐ ☐ ☐ ☐

On a scale of 1 to 5, would you buy this bottled water product?

Definitely would not purchase Definitely would purchase

☐ ☐ ☐ ☐ ☐

Sort the labels from least healthy to healthiest. Please register the codes according to your order:

_____ _____ _____ _____ _____ _____ _____ _____ _____

Sort the tags from the one you like the least to the one you like you like more. Please register the codes according to your ordination:

_____ _____ _____ _____ _____ _____ _____ _____ _____

DATA:

GENDER: F ☐ M ☐

AGE: 18-30 ☐ 31-40 ☐ 41-50 ☐ Over 50 ☐

PLACE: Costa ☐ Sierra ☐ Selva ☐

N.S.E: Low (1025-1500) ☐ Medium (1500-2000) ☐ High (2500-4000) ☐

Label evaluation report – Stage 2

Introduction:

welcome to this sensory evaluation of bottled water, in order to continue you must give your informed consent, remember that you can withdraw at any time you consider appropriate, any questions can be made to the person responsible for the sensory analysis (Reynaldo Silva-Paz or Tito Prada-Linares), wish to continue by accepting informed consent:

Yes ☐ No ☐

Indications:

- You will receive 04 water labels.
- Please look at the labels carefully and answer the following questions

Sample N°: __________

On a scale of 1 to 9, how much do you like this bottled water product?

I dislike it a lot I like it a lot

☐ ☐ ☐ ☐ ☐ ☐ ☐ ☐ ☐

On a scale of 1 to 5, would you buy this bottled water product?

Definitely would not purchase Definitely would purchase

☐ ☐ ☐ ☐ ☐

Sort the labels from least healthy to healthiest. Please register the codes according to your order:

__________ __________ __________ __________

Sort the tags from the one you like the least to the one you like you like more. Please register the codes according to your ordination:

__________ __________ __________ __________

DATA:

GENDER: F ☐ M ☐

AGE: 18-30 ☐ 31-40 ☐ 41-50 ☐ Over 50 ☐

PLACE: Costa ☐ Sierra ☐ Selva ☐

N.S.E: Low (1025-1500) ☐ Medium (1500-2000) ☐ High (2500-4000) ☐
